# Treatment Experience With Inhaled Corticosteroids in Combination with Remdesivir and Dexamethasone Among COVID-19 Patients Admitted to a Rural Community Hospital: A Case Series

**DOI:** 10.7759/cureus.11787

**Published:** 2020-11-30

**Authors:** Santosh Yatam Ganesh, Nagakrishnal Nachimuthu

**Affiliations:** 1 Internal Medicine, CHI St. Luke's Health - Memorial Hospital, Livingston, USA

**Keywords:** covid 19, infectious disease, inhaled steroids, remdesivir, dexamethasone convalescent plasma, pandemic era

## Abstract

Background

Pneumonia caused by severe acute respiratory syndrome coronavirus 2 (SARS-CoV-2) can cause severe inflammation of the lungs resulting in acute respiratory distress syndrome (ARDS). Current treatment guidelines support use of remdesivir as well as dexamethasone in hypoxic patients. There is very little information known about use of inhaled corticosteroids (ICS) in combination with the other two medications.

Methods and outcomes

We report our experience among six coronavirus disease 2019 (COVID-19) patients who received ICS, remdesivir and dexamethasone for treatment as well as their outcomes. Data were obtained from retrospective chart review during a two-week period from July 8, 2020 to July 22, 2020. Five patients were treated successfully and discharged home. One patient expired.

Conclusions

This case series highlights the possible benefits of inhaled steroids in treatment of COVID-19 patients with hypoxia. Further randomized controlled studies are needed to assess inhaled corticosteroids as possible treatment either alone or in combination with systemic steroids for treating COVID-19 patients. Dose and optimal duration need to be studied and evaluated.

## Introduction

Coronavirus disease 2019 (COVID-19) pneumonia caused by severe acute respiratory syndrome coronavirus 2 (SARS-CoV-2) was initially reported in China in late 2019 [1]. As of August 7th, 2020 there have been more than 17.5 million cases worldwide and more than 4.5 million cases in the United States [2]. It has been a rapidly evolving disease with frequent updates in treatment guidelines based on ongoing research. Therapeutic options available so far are minimal. In a randomized double-blinded control study, Adaptive COVID-19 Treatment Trial (ACTT-1), remdesivir was noted to reduce the recovery period in patients who were admitted to hospital [3]. Based on this data, remdesivir has been included as part of treatment in hypoxic patients admitted to the hospital who met certain institutional criteria. Early data from the RECOVERY trial reported mortality benefit among hypoxic and mechanically ventilated patients who received dexamethasone [4]. There is anecdotal evidence based on case reports and in vitro studies demonstrating the benefits of using inhaled corticosteroids (ICS) in suppressing replication of the virus and cytokine production [5-7]. We describe a case series of six patients with COVID-19 who were treated with ICS, remdesivir and dexamethasone and their outcomes.

## Materials and methods

This is an observational case series from a small hospital in rural Texas. Six patients admitted to the hospital with confirmed case of COVID-19 between July 8 and July 22, 2020 and received remdesivir, dexamethasone and ICS were included. These patients did not have a diagnosis of chronic obstructive pulmonary disease (COPD) or asthma prior to hospitalization and were not on ICS at home. We consulted our Institutional Review Board (IRB) for collecting data and they recommended obtaining individual patient consent since there were only six patients. No patient identifiable information was included. Data were obtained from review of electronic medical records. Each case describes patient symptoms, oxygenation status and outcomes. 

Laboratory data are given in Table 1.

**Table 1:** Laboratory data on admission ALT: alanine aminotransferase, AST: aspartate aminotransferase

Laboratory data	Case 1	Case 2	Case 3	Case 4	Case 5	Case 6
White-cell count (10^3^/uL)	9.1	8.06	24.0	5.2	6.6	7.8
Lymphocyte (%)	11.8	5.0	5.0	5.7	12.1	5.6
Platelets (10^3^/uL)	254	182	313	174	121	215
Creatinine (mg/dl)	0.7	1.5	1.2	0.8	0.9	0.8
AST (U/L)	37	76	35	83	78	22
ALT (U/L)	55	117	49	60	37	46
D-dimer (mg/L)	1.1	5.62	1.43	0.98	2.18	1.08
Ferritin (ng/ml)	403.4	134	445.4	233.6	1531	458.7
Serum lactate dehydrogenase (LDH) (U/L)	234	379	307	N/A	540	404
C-reactive protein (mg/dl)	59.6	7.9	54.6	59.3	139	182
Interleukin-6 (pg/ml)	N/A	16.8	N/A	19.1	36.4	78.8
Procalcitonin (ng/ml)	0.04	0.34	N/A	0.05	0.28	0.10
Creatine Phosphokinase (U/L)	146	N/A	192	N/A	53	104
Troponin-I (ng/ml)	<0.015	N/A	<0.015	N/A	<0.015	<0.015

Home medication list is provided in Table 2.

**Table 2:** Home medication list

Case 1	Case 2	Case 3	Case 4	Case 5	Case 6
Bupropion, Clopidogrel, Furosemide, Hydrocodone, Levetiracetam, Levothyroxine, Metaxalone, Rosuvastatin and Spironolactone.	Amlodipine, Aspirin, Clopidogrel, Gabapentin, Levofloxacin, Levothyroxine Losartan, Metformin, Metoprolol Milk of magnesium, Pantoprazole and Tizanidine.	Allopurinol, Amlodipine, Aspirin, Atorvastatin, Dexamethasone, Hydrochlorothiazide, Losartan and Ondansetron.	Amlodipine, Atorvastatin, Bupropion, Duloxetine, Furosemide, Hydrocodone, Lisinopril, Metformin, Metoprolol, Montelukast and Testosterone.	Ezetimibe, Finasteride, Furosemide, Latanoprost, Metoprolol, Omeprazole, Pravastatin, Tamsulosin and Tolterodine.	Atorvastatin, Lisinopril and Metformin.

Criteria for emergency use authorization (EUA) of remdesivir in our institution during that time period was as follows: hospital admission, lab confirmed infection with SARS-CoV-2 by positive reverse transcriptase polymerase chain reaction (RT-PCR), symptom onset less than 10 days, not a candidate or declined enrollment in an on-site remdesivir clinical trial, requiring supplemental oxygen more than or equal to 4L/min or on vapotherm or bilevel positive airway pressure (BiPAP). Exclusion criteria for remdesivir were improving clinical condition, mechanical ventilation, extracorporeal membrane oxygenation (ECMO), liver function test (LFT) more than 5 upper limit of normal, renal dysfunction with estimated glomerular filtration rate (eGFR) less than 30, hemodialysis, peritoneal dialysis, palliative/hospice care or expected life expectancy of less than six months. Dexamethasone was used on patients who were hypoxic and requiring oxygen. The patients received inhaled mometasone 220 mcg two puffs twice a day for ICS which was used in hypoxic patients with no altered mental status. 

## Results

Case 1

A 70-year-old female with history of peripheral arterial disease (PAD), hypertension, non-smoker presented to the emergency room (ER) with complaints of shortness of breath going on for four days. Patient was tested positive for COVID-19 as outpatient via nasopharyngeal swab for RT-PCR test. She was exposed to her husband who had tested positive for COVID-19. She had associated nonproductive cough and fever but denied any chest pain. On examination patient was noted to be hypoxic requiring 2L oxygen via nasal cannula. Lung examination did not reveal wheezing or crepitus. Chest x-ray did not show any evidence of acute cardiopulmonary disease. Treatment was started with dexamethasone. Overnight patient progressively became short of breath requiring 4L oxygen to keep oxygen saturation above 92%. Arterial blood gas (ABG) confirmed worsening hypoxia compared to admission ABG. Remdesivir was initiated. Patient was on high-dose deep vein thrombosis (DVT) prophylaxis with enoxaparin as D-dimer was noted to be elevated. Patient was started on inhaled mometasone on day two until discharge. After completing a five-day course of remdesivir patient was discharged home on room air on day six.

Case 2

**Figure 1:**
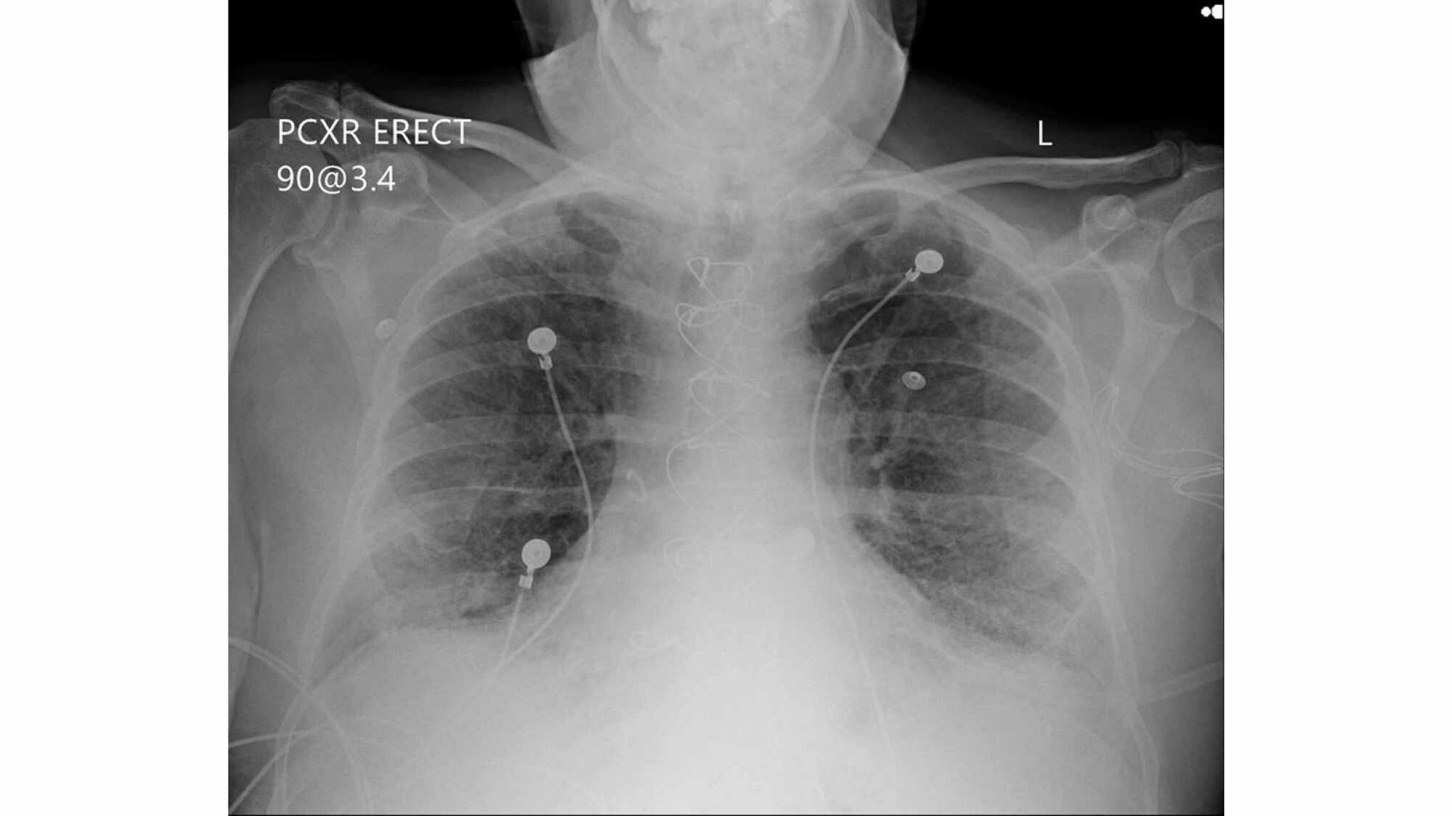
Bilateral diffuse interstitial opacities

A 70-year-old male with history of coronary artery disease, hypertension, diabetes mellitus, hypothyroidism, previous history of smoking presented to ER with complaints of shortness of breath going on for nine days. He had associated symptoms of productive cough, fatigue, fever and chills. Eight days prior to admission, patient was diagnosed with COVID-19 via nasopharyngeal swab for RT-PCR test. He worked at a health care facility and was exposed at work. This was his second visit to the ER. He saw his primary care physician and was noted to be hypoxic, so he was sent to the ER and he again tested positive for COVID-19. On examination he was on 6L oxygen via nasal cannula. Lung examination revealed bilateral rhonchi and diminished breath sounds. Chest x-ray exhibited increased left basilar opacities compared to the previous x-ray (Figure 1). Vancomycin and cefepime were initiated empirically. He was also started on remdesivir, however his condition progressively worsened and his oxygenation requirements increased rapidly. He continued to be in respiratory distress on non-rebreather (NRB) mask so was switched to BiPAP. Patient required 100% fraction of inspired oxygen (Fi02) on BiPAP to keep oxygen saturation above 90%. Due to elevated D-dimer he was started on high-dose enoxaparin for DVT prophylaxis. Mometasone inhalation was added on day two of admission. Convalescent plasma was transfused on day three. Patient’s condition further worsened, and he was intubated. Patient continued to require 100% FiO2 and positive end-expiratory pressure (PEEP) of 15 to maintain oxygen above 88%. With no improvement in his condition, patient’s family opted to make him do not resuscitate (DNR). He passed away on day 11.

Case 3

A 72-year-old male with history of hypertension, gout, obstructive sleep apnea and non-smoker presented to the ER with complaints of shortness of breath going on for one week. He tested positive for COVID-19 a week ago using nasopharyngeal swab for RT- PCR. He was exposed to his grandson who had tested positive for COVID-19. He had presented to the ER four days prior to this admission and was sent home on steroids and cough medication. He noticed worsening shortness of breath and noted his oxygen levels low on home pulse oximeter, so he came back to the ER. He tested positive again for COVID-19. On examination patient was noted to be in respiratory distress on non-rebreather mask and had to be placed on BiPAP. He had reduced breath sounds bilaterally. Chest x-ray revealed bilateral infiltrates. Remdesivir and dexamethasone were initiated along with empiric ceftriaxone and azithromycin. He was placed on enoxaparin for DVT prophylaxis. Convalescent plasma was transfused on day three as he continued to be hypoxic requiring 15L high flow nasal cannula. Mometasone inhalation was started on day one of admission. His condition slowly improved and he was discharged on day seven on 3L nasal cannula.

Case 4

**Figure 2:**
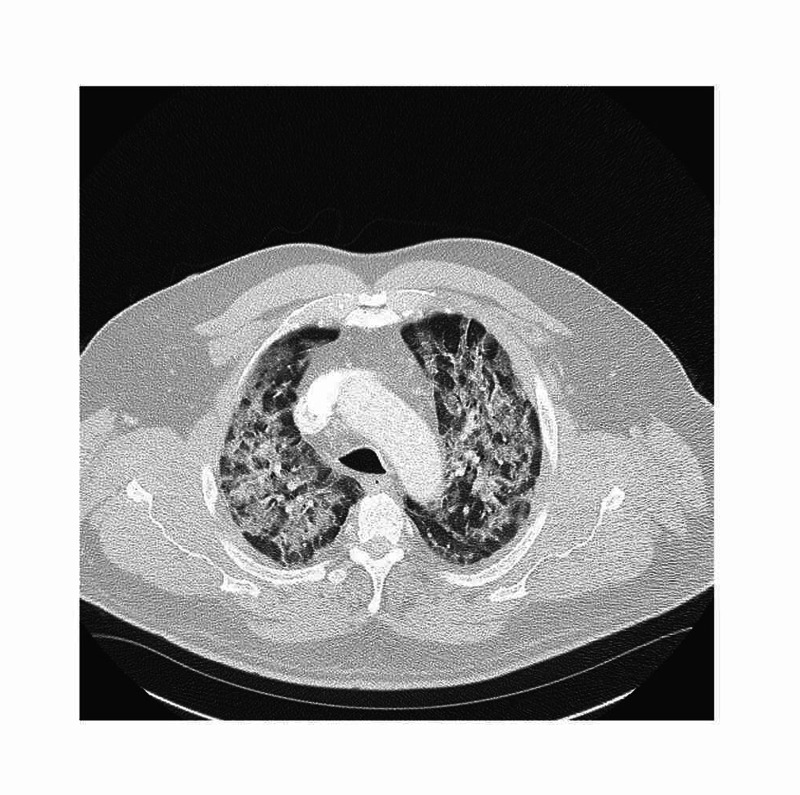
Extensive subpleural ground-glass opacities

A 66-year-old male with history of asthma, hypertension, diabetes mellitus, dyslipidemia and smoking presented to the ER with complaints of shortness of breath going on for one week. Patient was diagnosed with COVID-19 via nasopharyngeal swab RT-PCR a week prior due to exposure at work. Shortness of breath progressively worsened with associated fever, cough, and fatigue. He also had associated loss of sense of smell and taste and decreased appetite. On arrival patient was noted to be hypoxic. Decreased breath sounds were observed. Chest x-ray showed increased interstitial marking. CT angiogram was negative for pulmonary embolism however did reveal diffuse ground-glass opacities (Figure 2). Patient required 3.5L of oxygen on arrival which quickly worsened requiring 15L via high flow nasal cannula to keep the oxygen saturation above 92%. Patient was started on empiric azithromycin, remdesivir and dexamethasone. Day two he was started on mometasone inhalation. He was on enoxaparin for DVT prophylaxis. Patient improved well with the above treatment and he was discharged home on day six on room air.

Case 5

A 76-year-old male with history of hypertension, previous history of smoking presented to the hospital with complaints of shortness of breath going on for two days. He had associated fever, chills and non-productive cough. He did not have any known exposure to COVID-19 and was tested positive using RT-PCR. Physical examination revealed rhonchi bilaterally and was noted to be hypoxic requiring non-rebreather mask to keep oxygen saturation above 92%. Chest x-ray revealed basilar interstitial pattern. Cefepime and azithromycin were started empirically along with remdesivir and dexamethasone. He was on enoxaparin for DVT prophylaxis. He remained hypoxic on 10L oxygen through nasal cannula. He was started on mometasone inhalation on day five. Patient slowly improved and was discharged home on 4L nasal oxygen on day 13.

Case 6

A 54-year-old male with history of hypertension, diabetes mellitus, non-smoker presented from prison with complaints of shortness of breath going on for one week. Patient had associated cough and fever. He lived in a dorm and was exposed to a few others who had tested positive for COVID-19. He tested positive for COVID-19 using RT-PCR. On examination bilaterally decreased lung sounds were noted and he was hypoxic requiring 6L oxygen via nasal cannula. He soon deteriorated and had to be placed on BiPAP on 60% FiO2. Chest x-ray noted bilateral diffuse infiltrates. Ceftriaxone and azithromycin were started empirically along with remdesivir and dexamethasone. Mometasone inhalation was initiated on day one. Patient received convalescent plasma on day four. He was on enoxaparin for DVT prophylaxis. Patient improved and was discharged on day nine on 4L oxygen by nasal cannula.

Summary of the results

Our patients were predominantly male in the age group between 54-76 years. Majority of the patients had symptoms of COVID-19 going on for at least a week. Half of the patients were current or ex-smokers. Most common comorbidity was hypertension and none of the patients had diagnosis of COPD or asthma. None of the patients were on ICS prior to admission. ICS was initiated in the first couple of days except one patient who received on day five. They were continued until discharge or death. Hypoxia improved on all patients except one who presented sick to the hospital requiring high amount of oxygenation via nasal canula on admission. This patient quickly deteriorated requiring BiPAP because of profound hypoxia, later mechanically ventilated and passed away eventually. 

All patients but one had changes of bilateral infiltrates on chest X-ray. CT chest was done on one patient which revealed bilateral ground-glass opacities. Four patients had pneumonia panel and respiratory cultures which were negative for superimposed bacterial infection. Two patients did not have expectoration of sputum for pneumonia panel and sputum cultures. All patients had negative blood cultures.

Remdesivir was started on the patients when they met criteria. Based on the recent RECOVERY trial all patients requiring oxygen were placed on dexamethasone. Our patients received inhaled steroids within 48 hours except one patient. To avoid aerosolization patients were given inhaled steroids rather than nebulized steroids.

One patient ended up requiring invasive ventilation, all other patients were either on non-invasive ventilation or oxygen via nasal cannula and they improved in terms of their oxygenation by the end of hospital course. Three patients were discharged on oxygen via nasal cannula. Mean length of hospital stay was 8.5 days.

## Discussion

There are limited therapeutic options available for COVID-19 options so far. Remdesivir and dexamethasone (systemic steroids) have been approved for treatment [3,4]. The use of ICS in COVID-19 patients has not been well studied so far. There are no guidelines for or against the use of ICS. Also, no strong recommendations are available for continuation or discontinuation of ICS among patients who are on these medications on a chronic basis for COPD or asthma. Consensus is to continue ICS if patients have been on it as maintenance medication. In our case series none of our patients were on ICS at home. 

It is noted that the chronic lung conditions like COPD and asthma are not the most common comorbidities among patients with SARS-CoV-2 infection, which is similar to patients in our case series in which hypertension, diabetes and obesity were noted to be more prevalent [8]. One theoretical explanation for such lower prevalence of COPD or asthma among COVID-19 patients is chronic use of ICS. This is supported by some of the in vitro studies that noted ICS suppress replication of virus [5]. An in vitro study also noted that ICS decreased cytokine production [7]. The counter-argument for not using ICS comes from studies which have shown an increase in risk of infections like pneumonia and upper respiratory tract among COPD and asthma, who were on chronic ICS with or without combination of bronchodilators [9]. In some in vitro studies it was also noted that ICS led to decreased rhinovirus elimination and decrease in immune response to rhinoviral infection [10]. However so far, no peer-reviewed randomized clinical trials have been published for or against the use of ICS among COVID-19 patients. An attempt for systemic review to evaluate the use of ICS among COVID-19 patients by Halpin et al. showed no available publications to review [11]. In a small case series of three patients, reported improved outcomes in patients who were treated with ICS [12]. Similar findings were noted in a case report [6].

In a study by Matsuyama et al. it was noted that inhaled ciclesonide and mometasone has antiviral and anti-inflammatory properties [5]. In the past similar effects were seen with Middle East respiratory syndrome coronavirus (MERS-CoV) [13]. Recent evidence of improved mortality with dexamethasone among hypoxic COVID-19 patients has further heightened the role of steroids in treating the inflammation caused by the virus [4]. The principal cause of worsening lung condition as noted in studies is the inflammatory response elucidated by the virus leading to acute respiratory distress syndrome (ARDS) and cytokine storm [14]. The use of steroids helps in alleviating such inflammation. ICS may provide similar benefit with fewer side effects compared to systemic steroids because of minimal systemic absorption. We are the first case series to our knowledge to report the use of inhaled mometasone along with remdesivir and systemic steroids in COVID-19 patients. ICS provide an attractive option as it can be prescribed as an outpatient treatment. Further studies are required for determining the optimal dosage and duration of inhalation steroids with or without addition of systemic steroids in treating COVID-19 patients.

## Conclusions

Inhaled steroids may offer an additional treatment option for the treatment of COVID-19 patients. Further studies are needed to find if inhaled steroids can be used alone or in combination with steroids for the treatment of COVID-19 patients as outpatient and inpatient treatment.
